# Video-based gait analysis using pose estimation can quantify gait differences among non-frail, pre-frail, and frail older adults

**DOI:** 10.64898/2026.08.04.26359742

**Published:** 2026-08-07

**Authors:** Kaleb Burch, Jaya Hamkins, Laura McDaniel, Ana Raquel Castro e Costa, Zechen Yang, Jan Stenum, Andrew Pagliocchini, Crystal Szczesny, Jacqueline Langdon, Rama Chellappa, Peter M. Abadir, Ryan T. Roemmich

## Abstract

Frailty is a common consequence of aging that makes individuals increasingly susceptible to adverse health outcomes. Frailty screening can identify pre-frail and frail individuals to prescribe interventions or inform clinical decision making to prevent or slow additional frailty progression. Objective, scalable, and automated frailty assessments may expedite and improve clinical frailty screening. Here, we leveraged human pose estimation for video-based gait analysis in older adults who were non-frail, pre-frail, and frail. We focused on gait because slow walking speed is key diagnostic criteria of frailty, and many gait deviations are often observed in older adults with frailty. We collected videos of 68 older adults (25 non-frail, 25 pre-frail, 18 frail) walking at both self-selected and fast paces and used an established pose estimation-based gait analysis approach to measure and compare gait parameters across frailty statuses. Pose estimation-based step time measurements were strongly correlated with manual annotations (self-selected: R ^2^ =0.93, fast: R ^2^ =0.80) and showed tight Bland-Altman limits of agreement (self-selected: -0.082 to 0.052s, fast: -0.114 to 0.110s), establishing validity of this video-based gait analysis approach in older adults. We then identified a series of cross-sectional differences in spatiotemporal gait parameters among non-frail, pre-frail, and frail older adults, demonstrating that video-based gait analysis can be useful for measuring gait differences across frailty statuses. This study demonstrates the potential of video-based pose estimation for scalable gait tracking across frailty statuses in older adults.

## Full Text Availability

The license terms selected by the author(s) for this preprint version do not permit archiving in PMC. The full text is available from the preprint server.

